# Traditional medicine as a potential treatment for Flammer syndrome

**DOI:** 10.1007/s13167-017-0091-9

**Published:** 2017-05-08

**Authors:** Akiko Kikuchi, Yukihiro Shiga, Shin Takayama, Ryutaro Arita, Shigeto Maekawa, Soichiro Kaneko, Noriko Himori, Tadashi Ishii, Toru Nakazawa

**Affiliations:** 1 Department of Kampo Medicine, Tohoku University Hospital, 1-1 Seiryo-machi, Aoba ward, Sendai, 980-8574 Japan; 2 Department of Ophthalmology, Tohoku University Graduate School of Medicine, 1-2 Seiryo-machi, Aoba ward, Sendai, 980-8575 Japan; 3 Department of Advanced Ophthalmic Medicine, Tohoku University Graduate School of Medicine, 1-2 Seiryo-machi, Aoba ward, Sendai, 980-8575 Japan; 4 Department of Retinal Disease Control, Tohoku University Graduate School of Medicine, 1-2 Seiryo-machi, Aoba ward, Sendai, 980-8575 Japan

## Flammer syndrome and TSS, a Kampo medicine

Flammer syndrome is a primary vascular dysregulation that is associated with characteristic clinical symptoms and examination findings [1, 2]. Although most patients with Flammer syndrome do not develop unrelated or distinct disease states, normal-tension glaucoma (NTG) has been reported as an associated condition [1, 2]. Konieczka et al. hypothesized that Flammer syndrome is associated with multiple sclerosis and other diseases [1–3] and suggested that treatment for Flammer syndrome may be prophylactic against related diseases that result from vascular dysregulation [3]. Currently, standardized diagnostic criteria or treatment protocols for Flammer syndrome have not been established [1]. However, based on traditional Japanese (Kampo) medicine, the formula tokishakuyakusan (TSS) could potentially affect Flammer syndrome.

Kampo medicine was introduced to Japan about 1500 years ago and was derived from traditional Chinese medicine. Since then, Kampo medicine has been evolving according to Japanese character, physical constitution, environment, and circumstances. In Japan, TSS (Danggui Shaoyao San in Chinese) is primarily administered to women with gynecological disorders who experience a cold sensation in their extremities [4, 5]. According to the theory of Kampo medicine, TSS remedies blood deficiencies and improves blood circulation, as well as alleviates abnormal fluid retention in the body. Previous studies have indicated that TSS improves iron deficiency anemia [6–8] and leukorrhagia [9]. Moreover, it had been reported that TSS may increase cerebral blood flow [10, 11], reduce oxidative stress in the central nervous system [12], inhibit platelet aggregation [13], regulate thrombosis in endothelial cells [14], and relax vascular smooth muscle [15].

Table 1 shows the comparative clinical symptoms and signs between Flammer syndrome and the indications for use of TSS. The major characteristics of Flammer syndrome, which are more common in women, including low body mass index, cold extremities, and low blood pressure, are similar to the indications for use of TSS. In addition, other features that are not identified are described in Table 1.

**Table 1:** Comparative characteristics of clinical symptoms and signs in patients with Flammer syndrome and indications for the use of tokishakuyakusan

	Flammer syndrome	Indications for TSS use
Common features	Common in women	Common in women
	Low body mass index	Low body mass index
	Cold extremities	Cold extremities
	Low blood pressure	Low blood pressure
	Dizziness	Dizziness
	Reduced feeling of thirst	With or without thirst
	Feeling cold	Feeling cold
	Headaches	Headaches
	Tinnitus	Tinnitus
Other features	Migraines	Anemia
	Long sleep onset time	General fatigue
	Increased pain sensitivity	Edema
	Increased drug sensitivity	Numbness of extremities
	Good smell perception	Soft stool
	Reversible skin blotches	Menstrual irregularity
	Perfectionism	Dysmenorrhea
		Vaginal discharge

## Flammer syndrome and NTG

NTG is a major comorbid disease of Flammer syndrome [1, 2]. In patients with Flammer syndrome, there is an increased rigidity of retinal vessels and impaired autoregulation of the ocular blood flow. In patients with comorbid glaucoma and Flammer syndrome, additional signs are observed including disc hemorrhages, increased retinal venous pressure, and activation of retinal astrocytes [1, 2]. Although glaucoma is one of the leading causes of vision loss, reduction of intraocular pressure is the only proven approach to treatment. However, reducing the intraocular pressure alone does not prevent the progression of visual field loss in all patients, since impaired ocular circulation can also contribute to the progression of glaucoma [16, 17].

## Potential of TSS for treatment of NTG and Flammer syndrome

**Fig. 1:**
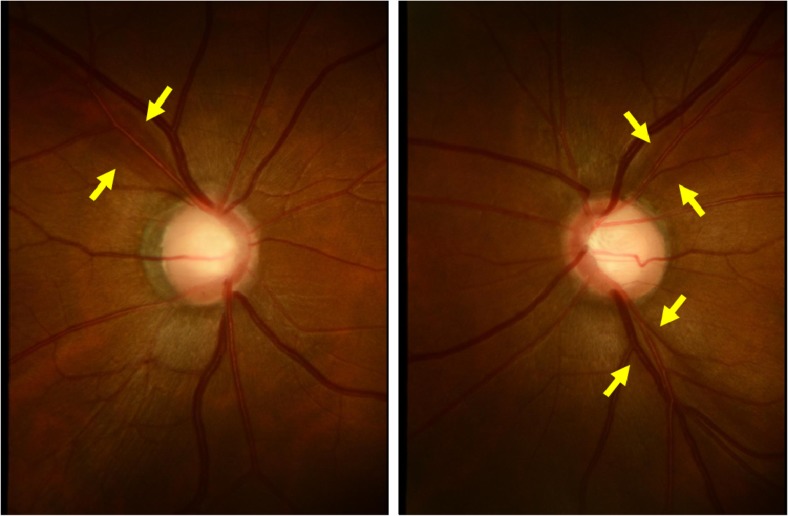
Fundus photograph of a representative case (*right*/*left*). Retinal layer defects were observed (*yellow arrows*)

**Fig. 2:**
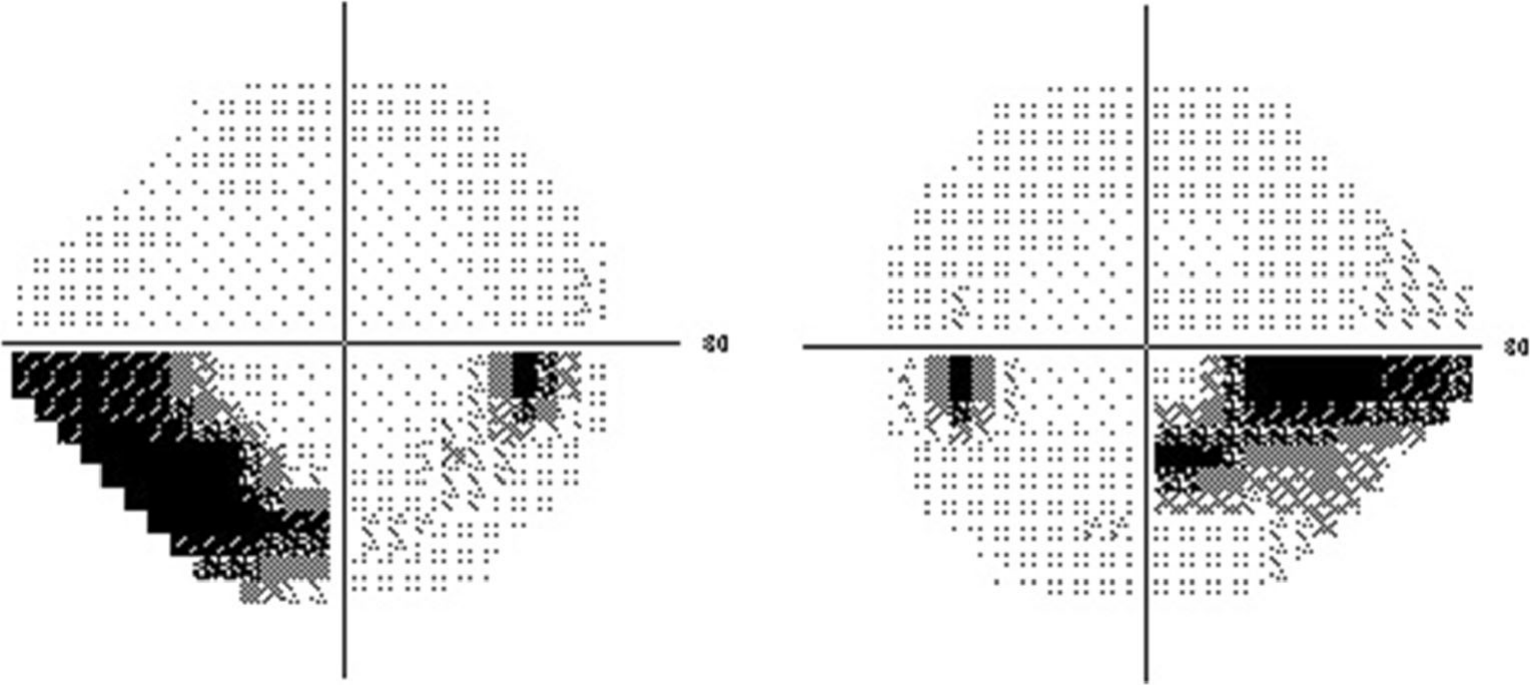
Visual field examination (*right*/*left*). A Humphrey 24-2 visual field test of the patient’s right eye shows an inferior-nasal defect. In addition, the visual field of the left eye shows inferior and upper-nasal defects associated with NTG

**Fig. 3:**
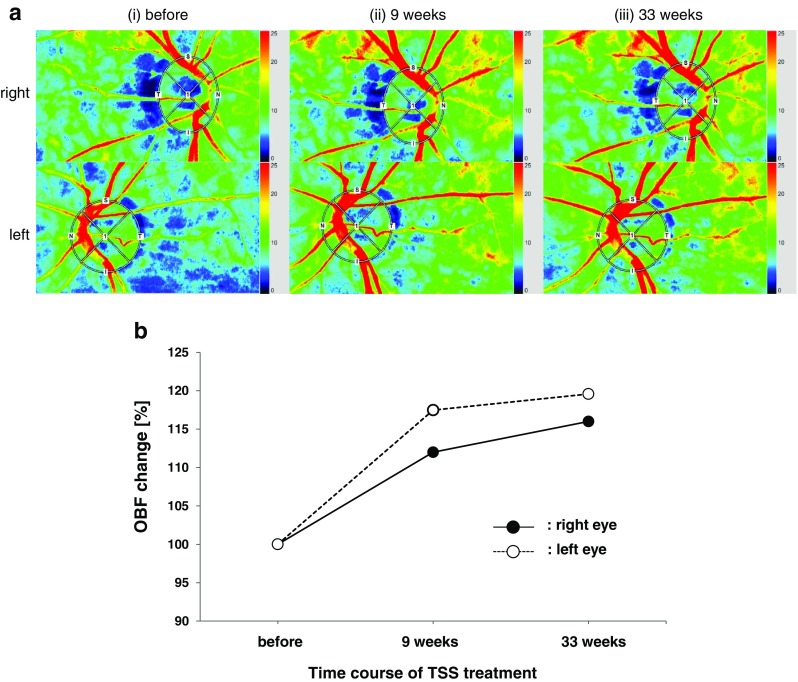
**a** Laser speckle flowgraphy images of the patients’ ocular circulation. The mean blur rate (MBR) images of the entire optic nerve head (ONH) before administration and at 9 and 33 weeks of TSS treatment (both eyes) (*i*): Composite blood flow map of the ONH before the administration of TSS. The MBR values are 12.5 (*right eye*) and 14.3 (*left eye*) (*ii*): Composite blood flow map of the ONH at 9 weeks of TSS treatment. The MBR values are 14.0 (*right eye*) and 16.8 (*left eye*) (*iii*): Composite blood flow map of the ONH at 33 weeks of TSS treatment. The MBR values are 14.5 (*right eye*) and 17.1 (*left eye*). **b** Dynamic changes in ocular blood flow (OBF) in response to TSS treatment. The rates of changes in ocular blood flow improved after 9 and 33 weeks, as compared to the pre-treatment measurements in both eyes

Recently, we reported that TSS increases ocular blood flow in healthy participants without decreasing blood pressure [18]. We are currently investigating whether TSS improves ocular blood flow in patients with NTG. Herein, we describe a representative case of a 42-year-old man with migraine and cold sensitivity who was diagnosed as having NTG in October 2013. His intraocular pressure was 16/16 mmHg without treatment. Figure 1 shows a photograph of the fundus at the patient’s first visit to our hospital, which indicates enlarged optic nerve cupping and retinal nerve fiber layer defects. Visual field defects were also detected in this patient (Fig. 2). We administered TSS extract granules (Tsumura and Co., Tokyo, Japan), at 7.5 g/d for 33 weeks, from February to September 2014. Following TSS treatment, the major symptoms of Flammer syndrome in the patient including sensitivity to cold and migraine frequency clearly improved. Figure 3 shows the changes in ocular blood flow measured using laser speckle flowgraphy of the optic nerve head. The mean blur rate, which is the relative ocular blood flow in arbitrary units, improved after 9 and 33 weeks, as compared to the pre-treatment measurement. The mean changes in visual field during treatment for TSS were +0.07 db/year in the right eye and −0.25 db/year in the left eye. The ocular pressure of the patient before TSS treatment, after 9 weeks, and after 33 weeks were 16, 14, and 14 mmHg in the right eye and 17, 14, and 14 mmHg in the left eye, respectively. Blood pressure and pulse rate at the same time intervals were 108/75/89, 103/69/81, and 106/72/80, respectively.

## Discussion

This case highlights that TSS can improve both the clinical symptoms of Flammer syndrome, as well as ocular blood flow, and prevent the progression of NTG. Visual field defects were not progressive during the entire TSS treatment duration. No change in the hemodynamics of the case due to the TSS treatment was found and was consistent with the result of our previous study [18]. We are currently conducting a study on a larger population to confirm these findings.

Impaired regulation of ocular blood flow is considered to be one of the causes of NTG. It has been reported that plasma endothelin-1 (ET-1) levels of NTG patients are significantly higher than those of healthy participants [19]. TSS extract granules weighing 7.5 g produced by Tsumura and Co. (Tokyo, Japan) contained 4.0 g Paeoniae Radix, 4.0 g Atractylodis Lanceae Rhizoma, 4.0 g Alismatis Rhizoma, 4.0 g Poria Sclerotium, 3.0 g Cnidii Rhizome, and 3.0 g Angelicae Radix. A previous study has reported that Alismatis Rhizoma and Poria Sclerotium inhibit the synthesis and expression of ET-1 in the glomeruli of nephritic rats [20]. Moreover, some studies have shown that TSS decreases ET levels in the ovary of rats but do not affect plasma ET levels [21, 22]. These studies suggest that TSS may inhibit the production of ET-1 at peripheral tissues without affecting ET-1 levels in plasma. However, no studies have evaluated the effects of TSS on ET-1 levels of retinal tissue and optic nerve head. Further study is needed to investigate the mechanism of the effect of TSS on ocular blood flow.

Recently, the concept of suboptimal health status (SHS) was advocated from the perspective of predictive, preventive, and personalized medicine [23, 24]. SHS is characterized by ambiguous health complaints and recognized as a subclinical, reversible stage of chronic disease. This perspective is highly associated with Flammer syndrome, which may contribute to the progression or development of chronic diseases.

## Limitation

The findings described herein are based on a single case; additional data from a case series are required to validate the efficacy of TSS. We have not yet investigated whether TSS statistically improves the clinical symptoms and signs of Flammer syndrome. Other common features may exist between Flammer syndrome and patients for whom TSS is indicated. Moreover, there are no reports on the frequency of Flammer syndrome in Japanese NTG patients. Further research is needed to determine the relationship between Flammer syndrome and TSS.

## Conclusion

Several common clinical features exist between subjects with Flammer syndrome and those for whom TSS is indicated. If TSS significantly increases the ocular blood flow in patients with NTG, TSS would be a potential treatment for patients with comorbid normal tension glaucoma and Flammer syndrome. Kampo medicine has been used for disease treatment, as well as for minor physical and mental health conditions. TSS may have a prophylactic effect in patients with Flammer syndrome who are at risk of developing related diseases.
